# Alternative methods to measure breast density in younger women

**DOI:** 10.1038/s41416-023-02201-5

**Published:** 2023-02-24

**Authors:** Rachel Lloyd, Sarah Pirikahu, Jane Walter, Gemma Cadby, Ellie Darcey, Dilukshi Perera, Martha Hickey, Christobel Saunders, Karol Karnowski, David D. Sampson, John Shepherd, Lothar Lilge, Jennifer Stone

**Affiliations:** 1 Genetic Epidemiology Group, School of Population and Global Health, The University of Western Australia, Perth, WA Australia; 2 University Health Network, Toronto, ON Canada; 3 Department of Obstetrics and Gynaecology, University of Melbourne and the Royal Women’s Hospital, Melbourne, VIC Australia; 4 Department of Surgery, Royal Melbourne Hospital, The University of Melbourne, Melbourne, VIC Australia; 5 Optical and Biomedical Engineering Laboratory School of Electrical, Electronic and Computer Engineering, The University of Western Australia, Perth, WA Australia; 6 Surry Biophotonics, Advanced Technology Institute and School of Biosciences and Medicine, The University of Surrey, Guildford, Surrey, UK; 7 Epidemiology and Population Sciences in the Pacific Program, University of Hawaii Cancer Center, Honolulu, HI USA; 8 Medical Biophysics, University of Toronto, Toronto, ON Canada

**Keywords:** Risk factors, Breast cancer, Epidemiology

## Abstract

**Background:**

Breast density is a strong and potentially modifiable breast cancer risk factor. Almost everything we know about breast density has been derived from mammography, and therefore, very little is known about breast density in younger women aged <40. This study examines the acceptability and performance of two alternative breast density measures, Optical Breast Spectroscopy (OBS) and Dual X-ray Absorptiometry (DXA), in women aged 18–40.

**Methods:**

Breast tissue composition (percent water, collagen, and lipid content) was measured in 539 women aged 18–40 using OBS. For a subset of 169 women, breast density was also measured via DXA (percent fibroglandular dense volume (%FGV), absolute dense volume (FGV), and non-dense volume (NFGV)). Acceptability of the measurement procedures was assessed using an adapted validated questionnaire. Performance was assessed by examining the correlation and agreement between the measures and their associations with known determinants of mammographic breast density.

**Results:**

Over 93% of participants deemed OBS and DXA to be acceptable. The correlation between OBS-%water + collagen and %FGV was 0.48. Age and BMI were inversely associated with OBS-%water + collagen and %FGV and positively associated with OBS-%lipid and NFGV.

**Conclusions:**

OBS and DXA provide acceptable and viable alternative methods to measure breast density in younger women aged 18–40 years.

## Background

Mammographic breast density, represented by the white radiographic appearance of epithelial and connective breast tissue on a mammogram, is one of the strongest predictors of breast cancer risk, with high breast density associated with increased risk [1, 2]. Breast density measures are highly correlated over time within women [3]. However, there is a large variation in breast density across women at all ages. Large twin studies have estimated that genetic factors are responsible for ~60% of the variation in breast density [4, 5], leaving ~40% of the variation to be explained by environmental/lifestyle factors. Age and body mass index (BMI) are the strongest predictors of breast density and explain between 7 and 15% of this variation when combined with reproductive factors [6]. Together, these data suggest that breast density is established at the time of breast formation, which is largely determined by genes, after which environmental factors act, on average, to decrease breast density as women age [3].

Breast density appears to be modifiable, and reducing breast density through medical intervention (e.g., tamoxifen) reduces breast cancer risk [7, 8]. Recent evidence suggests that doses as low as 2.5 mg are effective in reducing breast density and are well tolerated [9], providing a potential primary prevention strategy for women at high risk of breast cancer [10]. Younger women are an obvious target for prevention strategies since any prevention measure would need to start many years prior to the age of a potential diagnosis. However, little is known about breast density in younger women as mammography is not recommended for women under 40.

Bridging the gaps in knowledge regarding the distribution and determinants of breast density in younger women requires a safe, acceptable, and viable measurement method. Alternative methods include optical techniques [11–13] and Dual X-ray Absorptiometry (DXA). Previous versions of Optical Breast Spectroscopy (OBS; formerly referred to as Transillumination Breast Spectroscopy) [11] measure spectral differences in breast tissue composition using low-level visible and near-infrared light. Previously, our OBS data processing was based on comparing spectra shapes using principal component analysis [14, 15] and the principal component (PC) with the strongest correlation to water absorption was used as a representative measure of dense breast tissue. Water-associated absorption correlates to the fibroglandular tissue in the breast (i.e., the dense tissue) [16] and the representative PC has been shown to be highly correlated with mammographic breast density in screen-aged women (*r* = 0.88) [17]. Newly developed breast tissue chromophore concentrations have been derived through simulation [18]. For the current study, breast tissue composition using this new approach is calculated, providing measures of percent water (OBS-%water), percent collagen (OBS-%collagen), and percent lipid (OBS-%lipid). We also examine a combined measure of OBS-%water and OBS-%collagen (OBS-%water + collagen) as a measure of fibroglandular tissue [19].

Breast density can also be measured in younger women using DXA, which measures breast density using minimal X-ray radiation safely in non-pregnant individuals [20]. DXA measures percentage fibroglandular dense volume (%FGV), which has also been shown to be highly correlated with mammographic breast density in screen-aged women (*r* = 0.76) [18] and in pre-menopausal women (*r* = 0.72), with similar associations with age and BMI [21]. %FGV has also been shown to be higher in younger girls (aged 10–16), (median value 69.4%), compared to their mothers (median 35.8%) [22].

This study examines the acceptability and performance of these two alternative procedures, OBS and DXA, to measure breast density in younger women aged 18–40 years. We compare measures of OBS and DXA and investigate their associations with known determinants of mammographic breast density that also predict breast cancer risk.

## Methods

### Recruitment and epidemiological data

Five hundred and thirty-nine women aged between 18 and 40 were recruited via the University of Western Australia Crowd Sourcing website [23], Register4 [24], and word of mouth. Women who were previously diagnosed with breast cancer or had bilateral breast surgery (including mastectomy, lumpectomy, augmentation and reduction) were excluded. Pregnant women were unable to undergo DXA scans due to low-level radiation exposure.

Participation included a height measurement using a wall-mounted stadiometer and a weight measurement using digital floor weight scales, completion of an epidemiological questionnaire, an OBS breast scan, and a post-scan acceptability questionnaire. Areola size, skin colour and information regarding nipple piercings and scars/tattoos on the breasts were also recorded. Women recruited after October 2017 were also asked to complete a breast DXA scan.

BMI was calculated from the measured height and weight data (kg/m^2^). The epidemiological questionnaire included questions relating to hormonal contraceptive use (progesterone, combined, none), age of menarche, alcohol and tobacco use (former, never, current), pregnancy, age of first and last births, breastfeeding (former, never, current), and history of breast disease and family history of breast cancer (none, 1st degree, 2nd degree). The acceptability questionnaire assessed the overall acceptability of the OBS and DXA scans regarding scan comfort, duration, and positioning, using a 5-point Likert scale.

Breast density measurement data from contralateral breasts were used for women who had unilateral surgery and/or (self-reported) benign breast disease. Intraclass correlation coefficients (ICC) were used to assess intra- and inter-reader reliability for the DXA breast density measures.

Approval to conduct this research was provided by the University of Western Australia Human Research Ethics Committee in accordance with its ethics review and approval procedures (2020/ET000013). Informed written consent was obtained from all participants.

### Measuring breast density using OBS

**Fig. 1:**
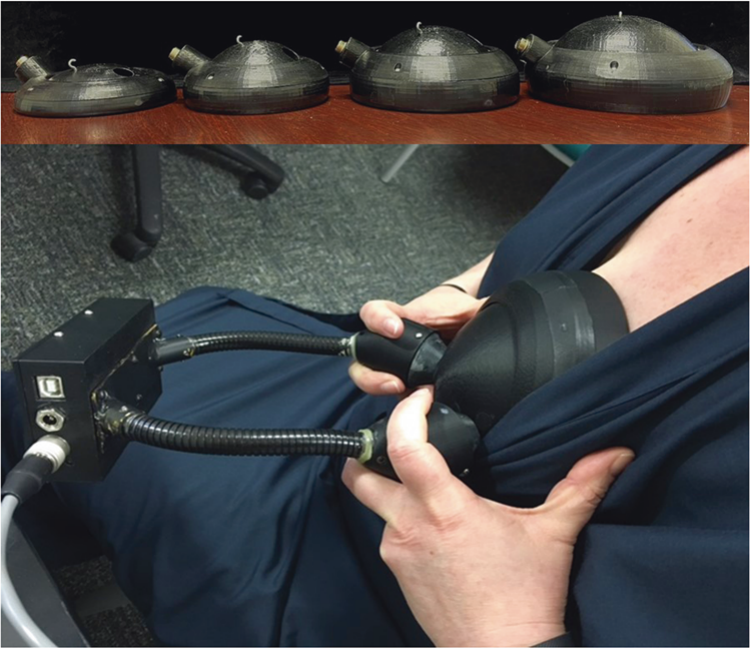
OBS cups and device. Photo demonstrating the four OBS measurement cup sizes in the top photo and the OBS device in use on a participant in the bottom photo.

Participants were asked to undress from the waist up and change into an open-fronted hospital gown for the examination. Participants chose an appropriate breast size from four cups representing approximate bra cup sizes A-D. If a woman’s breast exceeded the largest cup size, only a fraction of the volume would be optically interrogated. A trained research assistant performed a reference measure on a static silicone phantom mould using the chosen cup. Participants were asked to place the cup over their left breast and hold it in place during the scan. Figure 1 depicts the four OBS measurement cup sizes and the device in use. The scan took up to 5 min, depending on breast size. The process was repeated for the right breast followed by a second reference measurement and examined for repeatability [15]. A quality control check was performed immediately to determine whether a repeat scan was required.

Breast tissue composition measures using chromophore concentrations were calculated, providing measures of OBS-%water, OBS-%collagen and OBS-%lipid. A combined measure of %water plus %collagen (henceforth, OBS-%water + collagen) was also calculated, as was the principal component with the strongest water-associated absorption, used previously to represent the fibroglandular tissue in the breast (henceforth, OBS-PC3).

### Measuring breast density using DXA

DXA scans of both breasts were carried out using a clinical GE DXA machine according to the breast density measurement and calibration protocol previously outlined [25]. Briefly, participants were asked to remove jewellery and clothing from the waist up and change into an open-fronted hospital gown. Participants lay on their left side, positioning the left breast while holding the right breast out of frame during the scan before turning over to repeat on the opposite side. A repeat measure was done for the left breast. The scans took ~2 min per side.

The total projected breast area was manually delineated on each image, and the %FGV, absolute dense volume (FGV), and total breast volume were computed [20]. Non-dense volume (NFGV) was calculated by subtraction. Two independent readers measured each DXA scan, including repeated measurements of 50 scans to assess intra-reader reliability. Scans containing artefacts were excluded from the analysis. Repeated measurements of the left breast were compared, and those with differences in %FGV > 10% or FGV > 200cm^3^ were re-checked for image quality. An average of the left and right breast was used for each participant.

### Statistical analyses

#### Acceptability of OBS and DXA to measure breast density

Descriptive statistics (counts and percentages for categorical variables or means and standard deviations for continuous variables) were used to summarise participant characteristics and acceptability of OBS and DXA. Age at first and last birth were centred on their mean, and all women having never given birth were assigned 0.

#### Correlation and agreement between OBS and DXA measures

Scatterplots were used to visualise the correlation between OBS measures (%water, %collagen, %water + collagen, %lipids and OBS-PC3) and DXA measures (%FGV, FGV, NFGV). Corresponding Pearson correlation coefficients were estimated and stratified by OBS breast cup size. Linear mixed-effects models were used to investigate the interaction between cup size and a DXA measure as a predictor of an OBS measure. Likelihood ratio tests assessed evidence of an interaction. Agreement between quartiles and dichotomised measures of OBS and DXA were assessed using weighted Cohen’s Kappa statistics.

#### Determinants of OBS and DXA measures

Univariable and multivariable linear regression were used to investigate the associations of OBS measures with age, BMI, oral conceptive use, reproductive history, family history of breast cancer and smoking and alcohol use. Similarly, univariable and multivariable mixed-effect models were used to investigate the same associations with the DXA measures. The reader was treated as a random effect to account for repeated DXA measures (from two readers). Age and BMI were included in all multivariable models as these are known predictors for breast density, as was the number of live births when adjusting for breastfeeding. Diagnostic plots of age-adjusted residuals were checked for the model assumption of normality. This required FGV and NFGV to be square root transformed. Backward stepwise regression was performed for the multivariable models using a cut-off *P* value of <0.05. Model fit was compared using likelihood ratio tests and the Akaike information criterion. Sensitivity analysis was performed on women with both OBS and DXA measurements (i.e., removing women with OBS data only).

## Results

Of the 539 women who had an OBS scan, 8 were excluded post-examination due to ineligibility and 20 due to insufficient OBS data. Of the remaining 511 women, chromophore concentrations were estimated for 501 women, but the principal component analysis was only possible for 397 women.

Of the 169 women with DXA scans, 6 were excluded due to image artefacts, and 7 were excluded post-examination due to ineligibility. When DXA measures were the outcome, only women who had eligible OBS and DXA measures were included, leaving 132 women in the subset of women with DXA data. A flowchart showing recruitment and measurement numbers is presented in Supplementary Fig. S1.

Table 1 provides characteristics for the participants who have OBS measures and the subset who also have DXA measures, separately. The characteristics between both groups were very similar and over 90% were of European ethnicity. Women with OBS measures had an average age of 31.4 years (s.d. = 5.7) and a BMI of 25.4 (kg/m^2^, s.d. = 5.4). The majority of women with OBS measures had no live births (56.3%), had never breastfed (57.9%) and the mean age of menarche was 12.8 years (s.d. = 1.4). Most women had no family history of breast cancer (56.1%). The mean of OBS-%water+collagen was 38.2% (s.d. = 9.6) and the mean of %FGV was 45.2% (s.d. = 17.0).

**Table 1:** Table of characteristics for the participants with OBS measures (*N* = 501) and the subset with DXA measures (*N* = 132).

Characteristics	OBS chromophore (*N* = 501)	DXA (*N* = 132)
Age at questionnaire (s.d.)	31.4 (5.7)	31.0 (6.7)
Body mass index (kg/m^2^) (s.d.)	25.4 (5.4)	25.2 (5.1)
Ethnicity (%)		
European	463 (92.4)	120 (90.9)
Asian	23 (4.6)	5 (3.8)
South Asian	8 (1.6)	<5 (1.5)
Other	7 (1.4)	5 (3.8)
Ever been pregnant (%)		
Yes	253 (50.5)	66 (50.0)
No	248 (49.5)	66 (50.0)
Number of live births (%)		
0	282 (56.3)	72 (54.5)
1	63 (12.6)	12 (9.1)
2	102 (20.4)	28 (21.2)
3 or more	54 (10.8)	20 (15.1)
Age at first birth (s.d.)	28.6 (4.0)	27.9 (4.3)
Age at last birth (s.d.)	31.4 (3.4)	31.4 (3.9)
Ever or currently breastfeeding (%)		
Never	290 (57.9)	76 (57.6)
Former	172 (34.3)	50 (37.9)
Current	38 (7.6)	6 (4.6)
Missing	1 (0.2)	0 (0.0)
Currently using oral contraceptives (%)		
Yes	227 (45.3)	57 (43.2)
No	274 (54.7)	75 (56.8)
Active contraceptive (%)		
None	276 (55.1)	75 (56.8)
Progesterone	85 (17.0)	21 (15.9)
Combined	139 (27.7)	35 (26.5)
Missing	1 (0.2)	1 (0.8)
Age of menarche (s.d.)	12.8 (1.4)	12.6 (1.4)
Missing (%)	6 (1.2)	3 (2.3)
Family history of breast cancer (%)		
No history	281 (56.1)	76 (57.6)
1st degree	62 (12.4)	12 (9.1)
2nd degree	158 (31.5)	44 (33.3)
Benign breast disease—not removed (%)		
No	438 (87.4)	116 (87.9)
Yes—not removed	62 (12.4)	15 (11.4)
Missing	1 (0.2)	1 (0.8)
Smoking status (%)		
Never	410 (81.8)	106 (80.3)
Former	79 (15.8)	25 (18.9)
Current	12 (2.4)	<5 (0.8)
Alcohol consumption (%)		
Never	127 (25.3)	40 (30.3)
Former	117 (23.4)	25 (18.9)
Current	255 (50.9)	67 (50.8)
Missing	2 (0.4)	0 (0.0)
OBS cup size (%)		
1	141 (28.1)	42 (31.8)
2	125 (25.0)	24 (18.1)
3	155 (30.9)	47 (35.6)
4	80 (16.0)	19 (14.4)
Measurement: (s.d.)		
OBS-%water	18.4 (8.3)	19.9 (9.5)
OBS-%lipid	44.7 (11.2)	45.9 (13.5)
OBS-%collagen	19.9 (5.9)	19.0 (6.6)
OBS-%water + collagen	38.2 (9.6)	38.9 (11.0)
DXA %FGV	NA	45.2 (17.0)
DXA FGV (cm^3^)	NA	309.4 (128.6)
DXA NFGV (cm^3^)	NA	471.4 (347.2)

*s.d.* standard deviation, *OBS* optical breast spectroscopy, *DXA* dual X-ray absorptiometry, *%FGV* percent fibroglandular dense volume, *FGV* percent fibroglandular dense volume, *NFGV* non-dense volume, *sqrt* square root transformed.

The ICCs for %FGV, FGV and NFGV demonstrated high intra- and inter-reader reliability, with the ICCs > 0.90 for all three measures. For the repeated DXA scans on the left breast, the ICCs were likewise high, >0.85, for both %FGV and FGV.

### Acceptability of OBS and DXA measures

Over 93% of participants deemed the examination process for OBS to be an acceptable method of measuring breast density, compared with over 98% finding DXA to be acceptable. The exam comfort for both OBS and DXA was considered acceptable by more than 97% of participants. The duration of scans was also acceptable for both procedures (99% vs 98% for OBS and DXA, respectively. A stacked bar plot showing the results of six acceptability questions is presented in Supplementary Fig. S2.

### Correlation and agreement of OBS and DXA measures

**Fig. 2:**
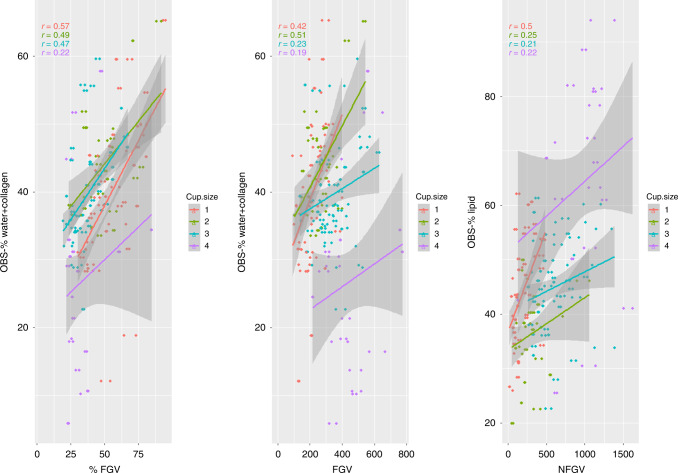
Correlations of OBS and DXA measures. Correlations (r) and scatterplots of DXA breast density measures (*x* axis) and OBS breast density measures (*y* axis), stratified by cup size (1-red; 2-green; 3-blue; 4-purple) for the 132 women who had both OBS and DXA scans. Solid lines represent linear fit from least squares regression; Dark grey shading represents the 95% Confidence Interval for each solid line. Panel 1: %FGV vs OBS-%water+collagen; Panel 2: FGV and OBS-%water+collagen; Panel 3: NFGV and OBS-%lipid.

Scatterplots and the corresponding correlations between OBS and DXA measures are shown in Fig. 2. Results of the linear regression indicated evidence of interaction between DXA measures and cup size when predicting most of the OBS measures. Therefore, we stratified each of the correlations by OBS cup size. Overall, the correlation coefficients for OBS-%water+collagen with %FGV and FGV were 0.48 and −0.015, respectively while the correlation between OBS-%lipids and NFGV was 0.48. The correlation coefficients for cup 4 were smaller and had broader 95% confidence intervals for all of the comparisons (0.22, 0.19 and 0.22, respectively).

Table 2 shows that the agreement between quartiles of %FGV and OBS-%water + collagen is fair, with weighted kappa scores of 0.40 (*P* = <0.001). The weighted kappa score for %FGV and FGV was −0.05 (*P* = 0.36), indicating poor agreement.

**Table 2:** Cross tabulation of %FGV quartiles with OBS-%water+collagen and FGV quartiles.

		OBS-%water+collagen quartiles				FGV quartiles			
		1st	2nd	3rd	4th	1st	2nd	3rd	4th
%FGV quartiles	1st	**15**	13	3	2	**5**	9	9	10
	2nd	12	**9**	6	6	11	**7**	8	7
	3rd	3	5	**18**	7	11	6	**8**	8
	4th	3	6	6	**18**	6	11	8	**8**

Perfect agreements are highlighted in bold.

### Determinants of OBS and DXA measures

Tables 3 and 4 summarise the regression results for OBS (OBS-%water+collagen and OBS-%lipids) and DXA measures (%FGV, %FGV, %NGFV) as the outcomes, respectively. Results for OBS-%water, OBS-%collagen and OBS-PC3 are presented in the Supplementary Figs. S3 and S4.

**Table 3:** Univariable and multivariable regression results for OBS breast density measures.

		OBS—percent water and percent collagen (*N* = 501)		OBS—percent lipid (*N* = 501)	
Characteristics	Categories	Univariable β (95% CI)	Multivariable β (95% CI)	Univariable β (95% CI)	Multivariable β (95% CI)
Age at questionnaire		−0.35 (−0.49, −0.20)***	−0.21 (−0.34, −0.08)**	0.36 (0.19, 0.53)***	0.23 (0.05, 0.41)**
Body mass index (kg/m^2^)		−0.76 (−0.90, −0.62)***	−0.53 (−0.69, −0.36)***	0.90 (0.74, 1.10)***	0.66 (0.48, 0.85)***
Ethnicity (vs. European)	Asian	3.14 (−0.84, 7.13)		−3.80 (−8.41, 0.81)	
	South Asian	3.21 (−3.45, 9.86)		−1.55 (−9.24, 6.15)	
	Other	−11.93 (−19.04, −4.82)***		17.86 (9.64, 26.08)***	
Ever pregnant (vs. no)	Yes	−2.53 (−4.20, −0.86)**		2.70 (0.70, 4.60)**	
Number of live births		−1.19 (−1.95, −0.44)**		1.32 (0.44, 2.20)**	0.46 (−1.02, 1.95)
Parity		−1.74 (−3.43, −0.04)*		1.90 (−0.08, 3.90)	
Age at first birth		0.08 (−0.26, 0.41)		−0.05 (−0.45, 0.35)	
Standardised		0.08 (−0.25, 0.40)		−0.05 (−0.43, 0.33)	
Age at last birth		−0.21 (−0.60, 0.17)		0.21 (−0.25, 0.67)	
Standardised		−0.21 (−0.58, 0.16)		0.21 (−0.22, 0.64)	
Benign breast disease not removed		1.48 (−1.07, 4.02)		−1.84 (−4.79, 1.12)	
Former or currently breastfeeding (vs. never)	Former	−2.10 (−3.92, −0.30)*		2.39 (−0.29, 4.49)*	−1.06 (−4.48, 2.37)
	Current	1.67 (−1.57, 4.91)		−3.06 (−6.83, 0.70)	−6.94 (−11.02, −2.85)***
Active contraception (vs. none)	Combined	1.54 (−0.43, 3.50)		−1.62 (−3.91, 0.66)	
	Progesterone	0.38 (−1.96, 2.73)		−0.46 (−3.18, 2.27)	
Age of menarche		0.76 (0.17, 1.35)**		−1.10 (−1.78, −0.41)**	
Family history (vs. none)	2nd degree	0.41 (−1.47, 2.29)		−0.41 (−2.60, 1.78)	
	1st degree	0.23 (−2.43, 2.88)		−0.82 (−3.91, 2.26)	
Smoking status (vs. never)	Former	−2.72 (−5.02, −0.42)*		3.08 (0.40, 5.75)*	
	Current	−6.66 (−12.14, −1.18)*		9.02 (2.65, 15.39)***	
Alcohol consumption (vs. current)	Former	−0.62 (−2.72, 1.48)		0.49 (−1.96, 2.94)	
	Never	−0.31 (−2.35, 1.73)		1.44 (−0.94, 3.81)	
OBS cup size (vs. 1)	2	1.47 (−0.63, 3.59)	2.44 (0.42, 4.47)*	−5.12 (−7.53, −2.71)***	−6.22 (−8.49, −3.95)***
	3	−2.89 (−4.90, −0.90)**	0.22 (−1.86, 2.31)	0.74 (−1.55, 3.02)	−2.91 (−5.24, −0.58)*
	4	−10.53 (−12.94, −8.12)***	−5.55 (−8.24, −2.87)***	11.09 (8.34, 13.84)***	5.47 (2.46, 8.49)***

Beta (β) represents the slope coefficient for the linear regression models. Backward stepwise regression was performed for the multivariable models using a cut-off *P* value of <0.05, missing cells indicate no evidence of association. Signif. codes: <=0.001***; <=0.01**; <=0.05*.

**Table 4:** Univariable and multivariable regression results for DXA breast density measures.

		Percent fibroglandular dense volume (*N* = 132)		Fibroglandular dense volume (cm^3^) (*N* = 132)		Non-dense volume (cm^3^) (*N* = 132)	
Characteristics	Categories	Univariable β (95% CI)	Multivariable β (95% CI)	Univariable β (95% CI)	Multivariable β (95% CI)	Univariable β (95% CI)	Multivariable β (95% CI)
Age at questionnaire		−0.92 (−1.21, −0.63)***	−0.58 (−0.82, −0.35)***	−0.03 (−0.10, 0.03)	−0.10 (−0.18, −0.02)**	0.35 (0.21, 0.49)***	0.18 (0.08, 0.29)***
Body mass index (kg/m^2^)		−2.24 (−2.55, −1.93)***	−1.94 (−2.24, −1.63)***	0.23 (0.15, 0.31)***	0.28 (0.20, 0.36)***	1.25 (1.13, 1.37)***	1.22 (1.10, 1.34)***
Ethnicity (vs. European)	Asian	5.94 (−4.84, 16.71)		−1.33 (−3.57, 0.90)		−3.69 (−8.66, 1.26)	
	South Asian	9.93 (−6.90, 26.76)		0.66 (−2.84, 4.15)		−4.14 (−11.89, 3.60)	
	Other	−9.49 (−20.27, 1.28)		1.52 (−0.71, 3.76)		7.87 (2.92, 12.83)	
Ever pregnant (vs. no)	Yes	−9.39 (−13.37, −5.41)***		−0.18 (−1.04, 0.67)		3.86 (1.99, 5.74)***	
Number of live births		−3.46 (−5.14, −1.79)***		0.05 (−0.31, 0.41)	0.04 (−0.64, 0.72)	1.64 (0.86, 2.42)***	−0.86 (−1.48, −0.23)**
Parity		−7.38 (−11.44, −3.32)***		0.09 (−0.77, 0.95)		3.15 (1.25, 5.06)***	
Age at first birth		0.45 (−0.19, 1.08)		−0.07 (−0.23, 0.09)		−0.28 (−0.62, 0.06)	
Standardised		0.59 (−0.11, 1.30)		−0.07 (−0.22, 0.08)		−0.34 (−0.67, −0.01)*	
Age at last birth		0.70 (−0.006, 1.40)		−0.005 (−0.18, 0.17)		−0.25 (−0.63, 0.13)	
Standardised		0.71 (−0.08, 1.50)		−0.005 (−0.17, 0.16)		−0.25 (−0.63, 0.12)	
Benign breast disease not removed		11.58 (5.20, 17.95)***	8.68 (4.01, 13.36)***	0.34 (−1.01, 1.68)		−4.06 (−7.04, −1.08)**	
Former or currently breastfeeding (vs. never)	Former	−7.87 (−11.99, −3.74)***		−0.79 (−1.66, 0.07)	−0.67 (−2.28, 0.95)	2.20 (0.21, 4.20)*	
	Current	14.45 (4.84, 24.06)**		3.54 (1.52, 5.56)***	5.37 (2.98, 7.76)***	−3.18 (−7.83, 1.47)**	
Active contraception (vs. none)	Combined	4.09 (−0.71,8.88)		0.26 (−0.75,1.27)		−1.62 (−3.87, 0.64)	
	Progesterone	−0.34 (−6.12, 5.45)		−0.002 (−1.22, 1.22)		1.27 (−1.45, 3.99)	
Age of menarche		1.79 (0.36, 3.23)**		−0.18 (−0.48, 0.13)		−1.09 (−1.76, −0.42)***	
Family history (vs. none)	2nd degree	2.74 (−1.72,7.21)	1.95 (−1.23,5.14)	−0.28 (−1.21, 0.65)		−1.33 (−3.43, 0.76)	
	1st degree	−6.32 (−13.65, 0.99)	−6.89 (−11.99, −1.78)**	−1.15 (−2.67, 0.38)		0.44 (−3.00, 3.89)	
Smoking status (vs. never)	Former	−11.10 (−16.20, 6.00)***	−5.44 (−9.41, −1.47)**	0.69 (−0.39, 1.78)		5.42 (3.06, 7.77)***	3.41 (1.84, 4.99)***
	Current	−20.48 (−43.51, 2.55)	4.52 (−12.44, 21.48)	3.48 (−1.43, 8.39)		14.73 (4.09, 25.37)**	2.43 (−4.29, 9.16)
Alcohol consumption (vs. current)	Former	0.45 (−5.08, 5.97)		−1.11 (−2.26, 0.03)*	−1.85 (−2.98, −0.90)***	−1.95 (−4.51, 0.62)	
	Never	5.38 (0.67, 10.10)*		−0.79 (−1.76, 0.19)	−1.31 (−2.90, −0.82)**	−3.09 (−5.28, −0.90)**	

Fibroglandular dense volume and non-dense volume were square root transformed. Multivariable mixed-effect models were used where the reader was treated as a random effect to account for repeated measures (from two readers). Backward stepwise regression was performed for the multivariable models using a cut-off *P* value of <0.05, missing cells indicate no evidence of association. Signif. codes: <=0.001***; <= 0.01**; <=0.05*.

### Age and BMI

Age was inversely associated with OBS-%water+collagen, FGV and %FGV and positively associated with OBS-%lipid and NFGV.

BMI was inversely associated with OBS-%water+collagen and %FGV and positively associated with OBS-%lipid and NFGV.

### Reproductive factors

Univariably, pregnancy variables (ever pregnant, number of live births, parity, and breastfeeding) were inversely associated with OBS-%water+collagen and %FGV. However, these associations were attenuated with the addition of age in the multivariable model.

No association was found between contraceptive use and the other OBS or DXA measures.

### Alcohol and smoking

Smoking was inversely associated with %FGV (*P*_trend_ = 0.01) and positively associated with NFGV (*P*_trend_ = <0.001). There was also evidence of the association between alcohol use and FGV (*P*_trend_ = <0.001).

### Family history

Incidence of 1st-degree family history of breast cancer was inversely associated with %FGV (*P*_trend_<0.01) however, this association was only observed in the multivariable model.

No evidence of association with family history was found for the OBS measures in the multivariable model.

### Other OBS outcomes

Similarly to OBS-%water+collagen, age and BMI were both negatively associated with OBS-%water and OBS-PC3. No evidence of association with age was found with OBS-%collagen, however, BMI was negatively associated and there was evidence a positive association with breastfeeding. Results are presented in Supplementary Table S1.

## Discussion

This study showed that OBS and DXA are acceptable methods for measuring breast tissue composition in younger women (18–40 years) and viable alternative breast density measures. We found that OBS and DXA measures are significantly correlated but less so in larger-breasted women. The determinants of OBS and DXA measures in younger women were found to be consistent with known determinants of mammographic breast density in screen-aged women, suggesting that much of what we know about the factors that influence breast density in older women can be extrapolated to younger women. Further investigation of OBS and DXA measures as predictors of breast cancer risk and their effectiveness in monitoring change in breast density over time is needed.

This study is the first to demonstrate participant acceptability of the OBS and DXA measurements, with over 93% of women reporting that both OBS and DXA procedures were acceptable. Finding a method of measuring breast density that is acceptable for women, particularly younger women, is vital to ensure high levels of participation for repeated measures and follow-up. OBS is a custom-designed modality specifically designed to quantify a breast density surrogate and is potentially a preferred procedure for repeated measures as it is much more cost-effective, portable, easy to use, and emits no ionising radiation, compared to DXA.

Overall, OBS%-water+collagen was correlated with %FGV (*r* = 0.48), consistent with recently reported correlations in adolescent girls [25]. Reported correlations between breast density measures vary significantly by modality (e.g., MRI, DXA, OBS, mammography), method (e.g., area vs. volume, automated vs. radiologist), and metrics (e.g., percent vs. absolute, continuous vs. categorical) [18–20, 26–29]. Unfortunately, there is no accepted correlation “threshold” that definitively validates one breast density measure compared to another. Nor is there a gold standard to compare alternative breast density measures in younger women, making definitive claims of validity challenging. We found that the correlations between OBS and DXA measures were stronger for smaller cup sizes, suggesting that it is harder to measure breast density accurately in larger-breasted women. Larger breast sizes might increase the probability of measurement error in both modalities. In particular, the OBS device was prone to under-sampling of the breast volume in large-breasted women resulting in a design improvement for the largest two cups, previously described in detail [22]. However, we currently do not have sufficient data in this study to thoroughly examine the impact of the design upgrade on improving the OBS-%water+collagen correlation with the %FGV and FGV measures. Another potential source of measurement error is that for some larger-breasted women, the entire breast did not sit entirely within the OBS cup. Therefore, the resulting chromophore concentrations may not reflect the entire breast and the ratio of the fibroglandular tissue to overall breast tissue may be increased. Unfortunately, the permissible light exposure to the skin, according to the American National Standards Institute (Z136.1-2007), does not currently allow for a device design with larger cups capable of measuring the entire volume in very large-breasted women.

We also assessed agreement between quartiles of OBS-%water+collagen and %FGV, which we found to be low. However, the agreement between %FGV and FGV was even lower. Like correlation estimates, agreement statistics do not infer validation when comparing measurement techniques. Both %FGV and absolute FGV (measured from mammography) are known to strongly predict breast cancer risk [30]; that is, an agreement between percentage and absolute measures can be low, but both measures are still strongly associated with breast cancer risk. That is because they are both measuring slightly different things—one adjusting for breast size, the other not. Similarly, when comparing OBS and DXA measures, both measure different components of dense breast tissue (water absorption vs. fibroglandular tissue) using different techniques (optical vs. x-ray properties). It is unknown which measure of breast tissue composition is most likely to predict breast cancer risk and establishing a gold standard would require long-term follow-up of a large cohort with baseline measures to establish association with future risk.

In the absence of a gold standard breast density measurement that is safe for younger women, reporting associations with other measures associated with mammographic breast density and/or breast cancer risk is arguably a better approach to infer performance as pseudo-breast density measures. We found the determinants of OBS breast density and DXA breast density in younger women to be consistent with known determinants of mammographic breast density in screen-aged women. Age is strongly negatively associated with both the OBS and DXA breast density measures and positively associated with the non-dense or “fatty” measures, which is consistent with evidence that mammographic breast density significantly decreases with age [31]. BMI was also a strong determinant of the density measures and consistent with literature that density decreases as BMI increases; however, this association was reversed for DXA absolute dense volume, consistent with previous literature [21, 32–34]. We found associations with reproductive factors were stronger for the DXA density measures than the OBS density measures, where they attenuated with the addition of age in the multivariable models. Overall, strong associations with known determinants of mammographic breast density, particularly age and BMI, suggests that breast density associations can be extrapolated to younger women using OBS and DXA breast density measures.

Our study is the first to examine the relationship between an optical measure of the percentage of breast collagen and image-estimated fibroglanduar volume. We found no correlation between OBS-%collagen and overall %FGV, which is consistent with literature however, a positive association between collagen fibre density and local percent density measures has been reported among women referred for biopsy [35]. Collagen may also play a key role in promoting tumour initiation and metastasis. An increase in local mammographic breast density associated with increased collagen could indicate local tissue changes surrounding benign breast disease and breast cancer [36]. Taroni et al. reported a negative association between an optical measure of the percentage of breast collagen and age, BMI, and menopausal status and a positive association with the Breast Imaging Reporting and Data System category [19]. Our study observed an association between OBS-%collagen and BMI in the same direction as Taroni et al. but no association with age. Based on unpublished data, we have observed that changes in OBS-%collagen do not occur gradually over time but rather suddenly upon the onset of menopause, consistent with the findings of Taroni et al., and may explain why no association was seen in this study of younger pre-menopausal women. Optical tools like OBS provide a non-invasive method to measure breast collagen within large-scale epidemiological studies to further examine the role of collagen and breast cancer development.

This study also demonstrated that measuring breast density using chromophore concentrations is more effective/efficient compared to principal component measures. The number of usable observations was reduced for OBS-PC3 due to data processing. Less than 2% of available data was lost using chromophore concentration processing, instead of almost 25% lost using principal component analysis due to the stricter data requirements.

Study limitations include restrictions to participation due to breast size, effectively eliminating women with breasts larger than the biggest cup. Study strengths include its size, the largest investigation of breast density in younger women aged 18–40 to date, with over 500 participants. The inclusion of both optical and image-based measures enabled comparisons not previously reported within this age range.

## Summary

This study showed that OBS and DXA are acceptable and viable alternative methods of measuring breast density in younger women (18–40 years). OBS and DXA measures correlate but do not necessarily measure the same breast tissue composition. We presented new evidence regarding the determinants of breast density in younger women, suggesting that much of what we know about the factors that influence breast density in older women can be extrapolated to younger women. This has important implications for future research investigating the utility of measuring breast density in younger women to identify and target those at increased risk of breast cancer later in life. Our research focus will now shift to investigating the effectiveness of OBS and DXA to measure changes in breast density over time, informing the utility of breast density to monitor the effectiveness of breast cancer prevention strategies that target breast density reduction.

## Supplementary information

Supplementary Information

## Data Availability

The data supporting this study’s findings are available on request from the corresponding author. The data are not publicly available due to privacy or ethical restrictions.

## Code availability

Computer code used to generate results can be made available on request.
