# Supplementary material for: Alternative methods to measure breast density in younger women
Source: Br J Cancer. 2023 Feb 24;128(9):1701–9. doi: 10.1038/s41416-023-02201-5 (PMC10133329; doi:10.1038/s41416-023-02201-5)
Supplement: Supplementary file 1 — Supplementary Information [file 41416_2023_2201_MOESM1_ESM.docx]

**An alternative method to measure breast density in younger women**

Supplementary Information

Submitted is a single word document containing a flow chart showing recruitment and measurement, a stacked bar plot demonstrating participant acceptability, a table showing univariable and multivariable regression results for OBS-%water, OBS-%collagen and OBS-PC3 and plots showing the correlation between OBS-%water, OBS-%collagen and OBS-PC3 and %FGV and FGV.

539 women had an OBS assessment and completed epidemiological and an acceptability questionnaire

519 women had usable spectra data
(20 women excluded due to insufficient reference measurement data)

511 women met the study eligibility criteria

501 women had usable OBS chromophore data after processing

397 women usable PCA data available (i.e. ≥ 6 wavelengths per spectra to facilitate PCA)

132 women had a DXA assessment

*Figure S1: Flow chart showing recruitment and measurement numbers for the OBS and DXA assessments*

**
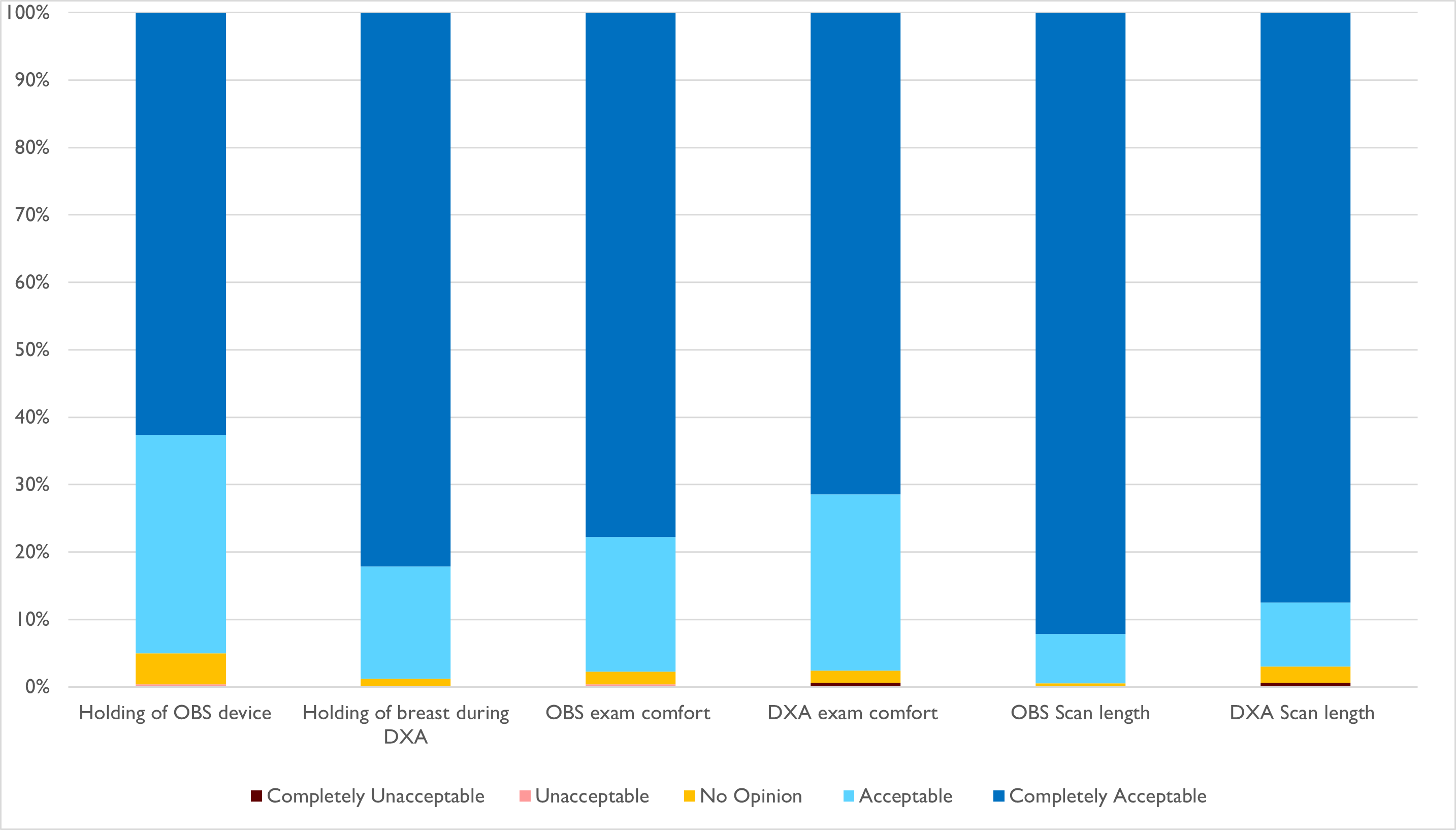
**

*Figure S2: Stacked bar plot showing results of six acceptability questions (x-axis) and acceptability as a percentage (y-axis). Questions were asked using a Likert scale with five response options: Completely Acceptable, Acceptable, No Opinion, Unacceptable, and Completely Unacceptable for 539 women that had OBS scans and 169 that had DXA scans.*


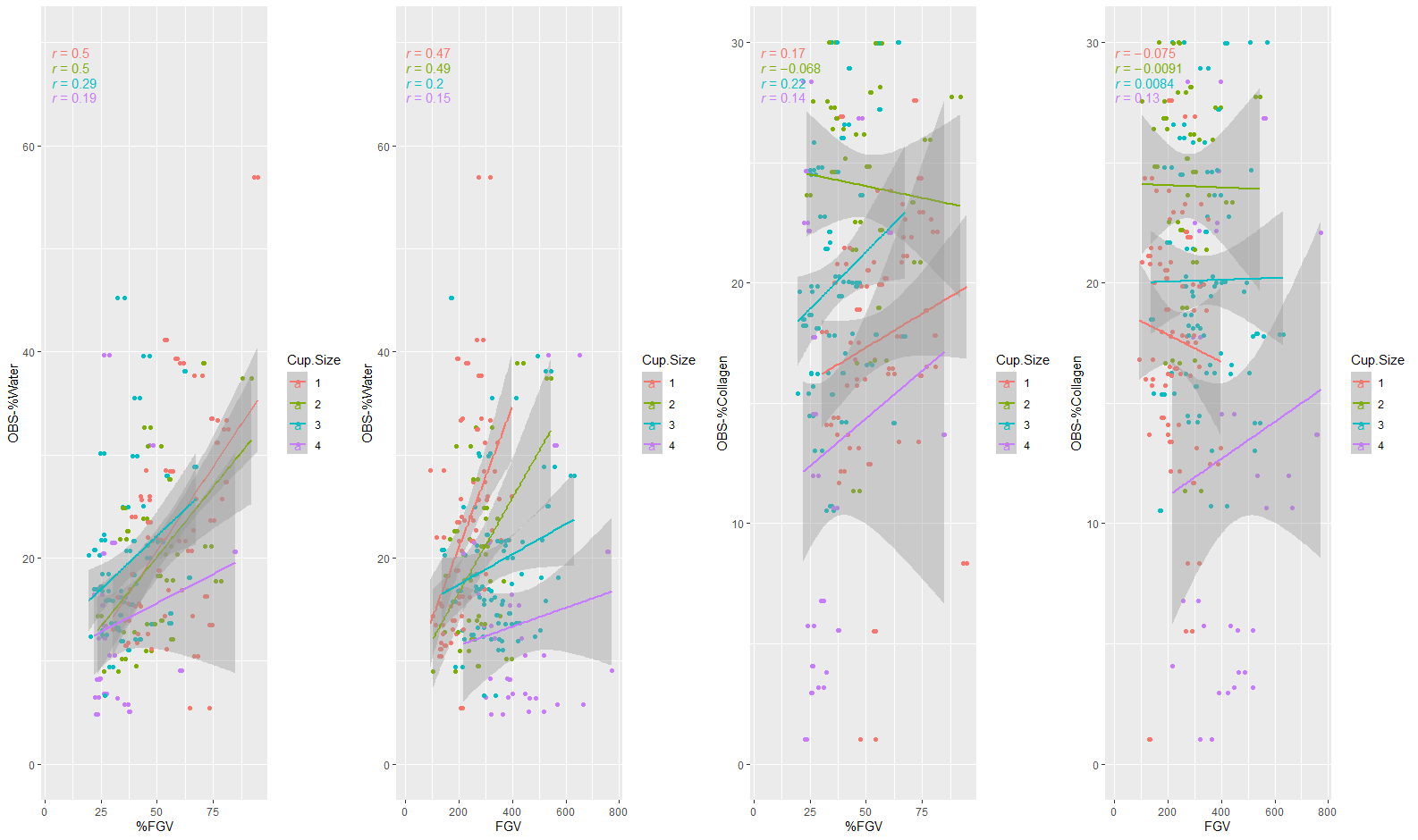


*Figure S3: Correlation between DXA breast density measures on the x-axis and OBS breast density measures on the y- axis breast density measures stratified by cup size*


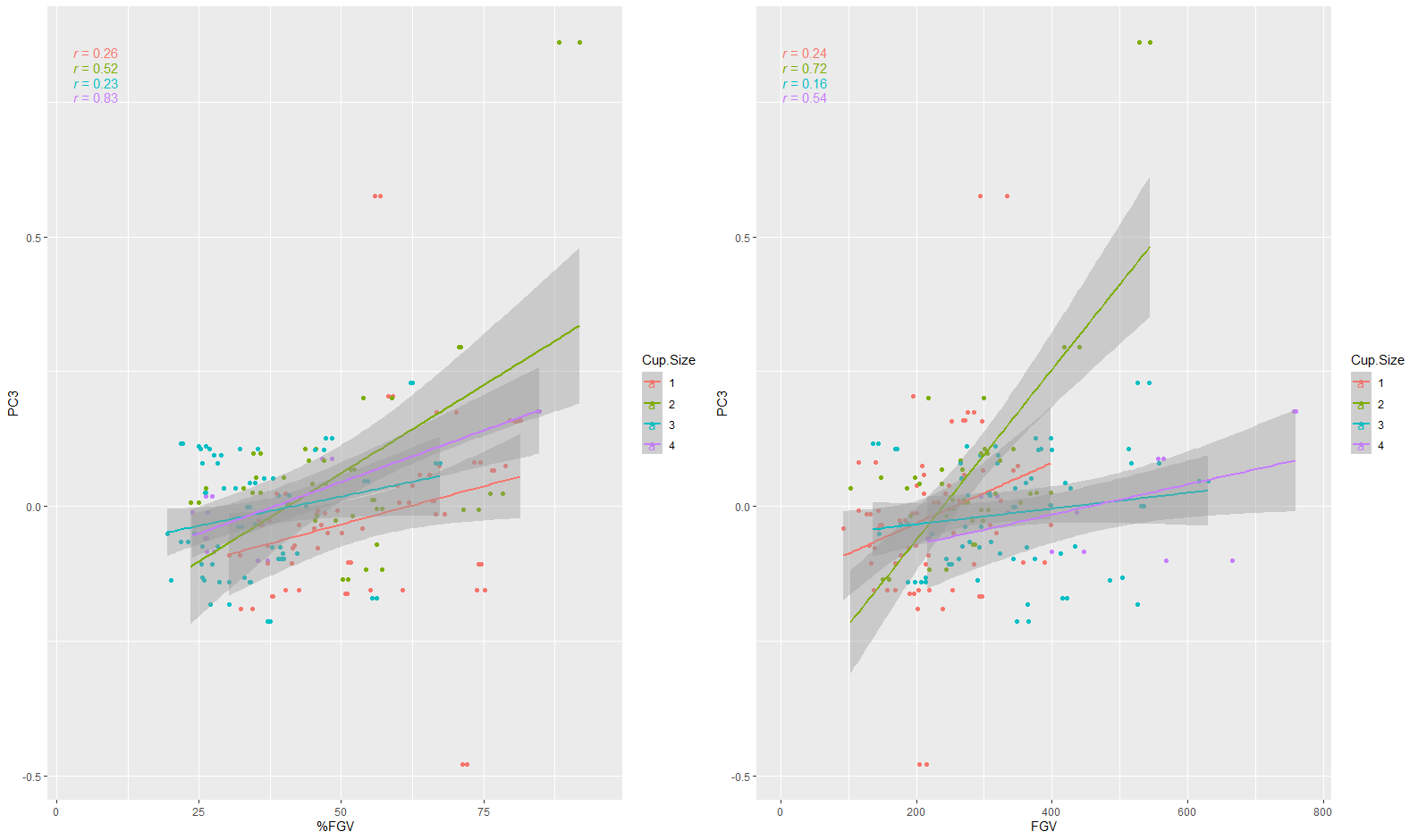


*Figure S4: Correlation between DXA breast density measures on the x-axis and OBS PC3 on the y- axis breast density measures stratified by cup size*

|  |  | OBS – Percent Water (N=501) | | OBS – Percent Collagen (N=501) | | OBS – PC3 (N=397) | |
| --- | --- | --- | --- | --- | --- | --- | --- |
| Characteristics | Categories | Univariable  β (95% CI) | Multivariable  β (95% CI) | Univariable  β (95% CI) | Multivariable  β (95% CI) | Univariable  β (95% CI) | Multivariable  β (95% CI) |
| Age at questionnaire |  | -0.39 (-0.51,-0.27)*** | -0.29 (-0.41,-0.18)*** | 0.04(-0.05,0.13) | -0.004(-0.10,0.09) | -0.003(-0.005,-0.001)** | -0.003(-0.005, 0.0005)* |
| Body Mass Index (kg/m2) |  | -0.53(-0.65,-0.40)*** | -0.28(-0.43,-0.14)*** | -0.23(-0.33,-0.14)*** | -0.22(-0.32,-0.12)*** | -0.004(-0.007,-0.002)*** | -0.004(-0.006,-0.002)*** |
| Ethnicity (vs. European) | Asian | 2.39 (-1.16,5.73) |  | 0.86(-1.59,3.31) |  | -0.01 (-0.04,0.06) |  |
|  | South Asian | 1.54(-4.22,7.29) |  | 1.67(-2.43,5.77) |  | 0.08(-0.01,0.17) |  |
|  | Other | -5.09(-11.24,1.06) |  | -6.84(-11.22,-2.47)** |  | 0.03(-0.10,0.14) |  |
| Ever Pregnant (vs. No) | Yes | -4.13(-5.53,-2.73)*** |  | 1.59(0.57,2.62)** |  | -0.03(-0.05,-0.003)* |  |
| Number of Live Births |  | -1.63(-2.26,-1.00)*** |  | 0.44(-0.03,0.90) | -0.41(-1.22,0.41) | -0.01(-0.02,0.001)* |  |
| Parity |  | -3.60(-5.00,-2.15)*** |  | 1.83(0.80,2.86)*** |  | -0.02(-0.04,0.01) |  |
| Age at first birth |  | -0.12(-0.37,0.13) |  | 0.20(-0.01,0.40) |  | 0.002(-0.003,0.007) |  |
| Standardised |  | -0.12(-0.40,0.16) |  | 0.20(-0.001,0.39) |  | 0.002(-0.003,0.007) |  |
| Age at last birth |  | -0.28(-0.56,0.004) |  | 0.07(-0.17,0.30) |  | -0.003(-0.009,0.003) |  |
| Standardised |  | -0.28(-0.59,0.04) |  | 0.07(-0.16,0.29) |  | -0.003(-0.009,0.003) |  |
| Benign breast disease not removed |  | -0.17(-2.37,2.02) |  | 1.65(0.10,3.21)* |  | -0.0006 (-0.04,0.04) |  |
| Former or currently breastfeeding (vs. Never) | Former | -3.36(-4.89,-1.84)*** |  | 1.25(0.17,2.33)* | 2.27(0.40,4.14)* | -0.02(-0.04,0.01) |  |
|  | Current | -4.02(-6.76,-1.29)** |  | 5.69(3.76,7.63)*** | 6.81(4.57,9.04)*** | 0.003(-0.05,0.06) |  |
| Active contraception (vs. None) | Combined | 1.66(-0.02,3.34) |  | -0.13(-1.33,1.08) |  | 0.005(-0.02,0.03) |  |
|  | Progesterone | 0.58(-1.43,2.58) |  | -0.19(-1.63,1.25) |  | 0.007(-0.03,0.04) |  |
| Age of Menarche |  | 0.38(-0.13,0.89) |  | 0.38(0.02,0.74)** |  | -0.0004(-0.009,0.008) |  |
| Family History (vs. None) | 2^nd^ Degree | 0.29(-1.33,1.90) |  | 0.12(-1.03,1.28) |  | 0.017(-0.01,0.04) |  |
|  | 1^st^ Degree | 0.78(-1.49,3.10) |  | -0.55(-2.18,1.07) |  | -0.003(-0.04,0.03) |  |
| Smoking Status (vs. Never) | Former | -2.38(-4.36,-0.40)** |  | -0.34(-1.76,1.08) |  | -0.009(-0.04,0.03) |  |
|  | Current | -3.75(-8.50,0.96) |  | -2.91(-6.30,0.48) |  | -0.06(-0.14,0.02) |  |
| Alcohol consumption (vs. Current) | Former | -1.81(-3.60,-0.02)* |  | 1.19(-0.10,2.47) |  | -0.01 (-0.04,0.02) |  |
|  | Never | 0.48(-1.25,2.22) |  | -0.79(-2.05,0.46) |  | 0.008(-0.02,0.04) |  |
| OBS Cup Size (vs. 1) | 2 | -4.12(-5.96,-2.27)*** | -3.50(-5.29,-1.71)*** | 5.59(4.29,6.89)*** | 5.75(4.51,6.99)*** | 0.06(0.03,0.09)*** |  |
|  | 3 | -4.89(-6.64,-3.14)*** | -2.85(-4.69,-1.01)** | 1.99(0.76,3.22)** | 2.84(1.56,4.11)*** | 0.01(-0.02,0.04) |  |
|  | 4 | -9.46(-11.56,-7.35)*** | -6.39(-8.76,-4.03)*** | -1.07(-2.56,0.41) | 0.19(-1.46,1.84) | 0.03(-0.02,0.09) |  |

*Table S1: Univariable and Multivariable regression results for OBS breast density measures. Beta (β) represents the slope coefficient for the linear regression models. Backwards stepwise regression was performed for the multivariable models using a cut off p-value of <0.05, missing cells were not significant. Signif. codes: <= 0.001 *** ; <= 0.01 **; <= 0.05 **
